# Ultrastructural studies on dengue virus type 2 infection of cultured human monocytes

**DOI:** 10.1186/1743-422X-2-26

**Published:** 2005-03-31

**Authors:** Jesus A Mosquera, Juan Pablo Hernandez, Nereida Valero, Luz Marina Espina, German J Añez

**Affiliations:** 1 Seccion de Inmunologia y Biologia Celular, Instituto de Investigaciones Clinicas "Dr. Americo Negrette". Facultad de Medicina, Universidad del Zulia, Maracaibo, Venezuela; 2 Instituto de Investigaciones Biologicas. Facultad de Medicina, Universidad del Zulia, Maracaibo, Venezuela; 3 Seccion de Virologia, Instituto de Investigaciones Clinicas "Dr. Americo Negrette". Facultad de Medicina, Universidad del Zulia, Maracaibo, Venezuela

## Abstract

**Background:**

Early interaction of dengue virus and monocyte/macrophages could be an important feature for virus dissemination after its initial entry via the mosquito vector. Since ultrastructural analysis of this interaction has not been reported, dengue type 2 (DEN2) virus-infected human monocyte cultures were studied at 1, 2, 4 and 6 hours after infection.

**Results:**

Typical dengue particles and fuzzy coated viral particles were 35 to 42 nm and 74 to 85 nm respectively. Viruses were engulfed by phagocytosis and macropicnocytosis leading to huge vacuoles and phagosomes inside the monocytes. Interaction of monocytes with DEN2 virus induced apoptosis, characterized by nuclear condensation and fragmentation, cellular shrinkage, blebbing and budding phenomena and phagocytosis of apoptotic cells by neighboring monocytes. This finding was confirmed by TUNEL. Ultrastructural features associated to DEN2 virus replication were not observed.

**Conclusion:**

These data suggest that clearance of the virus by monocytes and cellular death are the main features during the initial interaction of DEN2 virus and monocytes and this could be important in the rapid elimination of the virus after infection by mosquito vector.

## Background

Monocyte/macrophages are one of the major target of dengue virus and responsible for virus dissemination after its initial entry via the mosquito vector [1-3]. A detailed study of this early virus-monocyte interaction by electron microscopy has not been performed. Since ultrastructural study is one of the important analysis in the interaction virus-cell, we performed electron microscopy studies in DEN2 virus- infected human monocytes at 1, 2, 4 and 6 hours of culture, in order to get more information regarding to morphological aspects of virus, virus replication, cellular alterations and apoptosis.

## Results and discussion

### Virus particles

**Figure 1:**
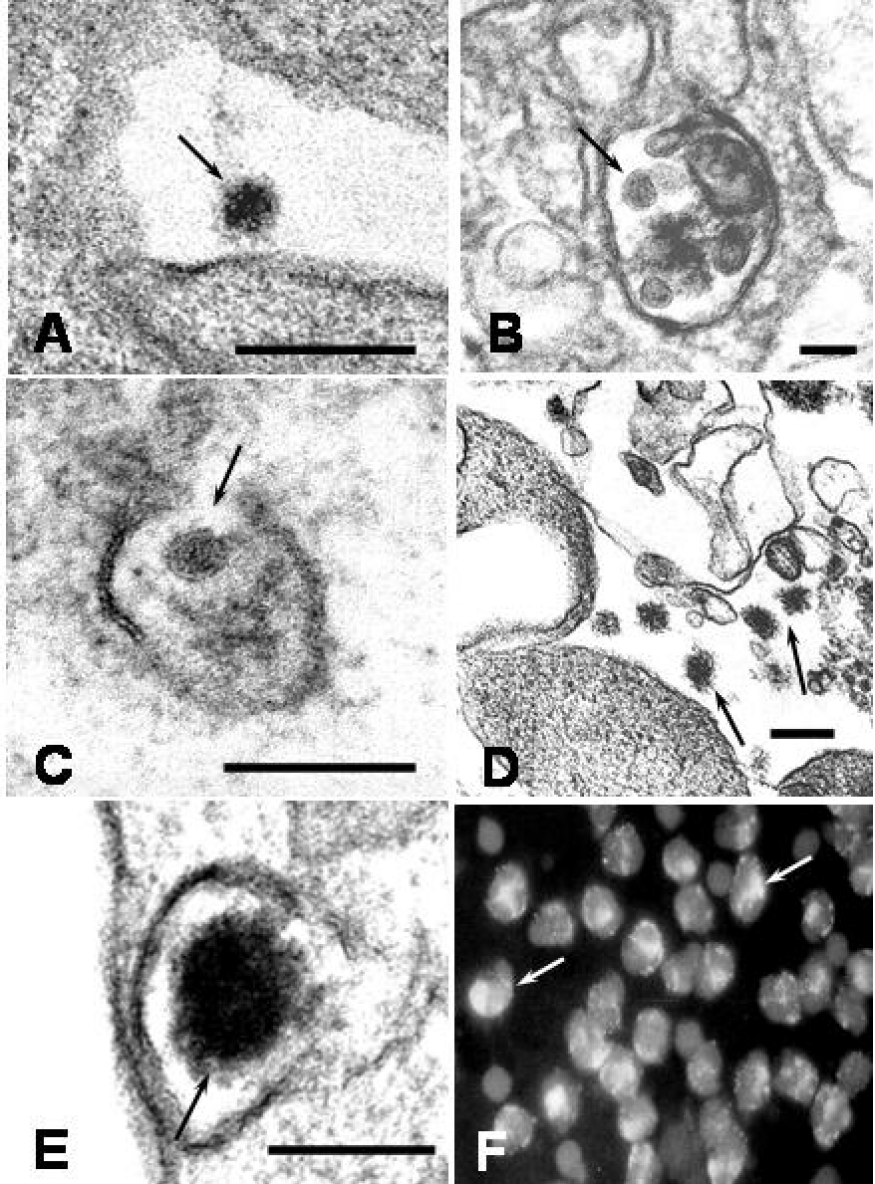
Electron microscope morphological observations of DEN2 virus particles. A) Typical viral particle in the extracellular environment (arrow; bar: 200 nm). B) Viral particles engulfed in an intracytoplasmic vacuole (arrow; bar: 50 nm). C) Membrane disruption of a vesicle containing a virus (arrow; bar: 100 nm). D) Fuzzy coated viral particles occur in the extracellular space (arrows; bar: 200 nm) E) A fuzzy coated viral particle showing an envelope with projections (arrow; bar: 100 nm). F) Immunofluorescence staining of DEN2 viral antigens at 4 h of culture. A diffuse and patchy pattern of fluorescence was observed in the cytoplasm (arrows). × 1000.

**Figure 2:**
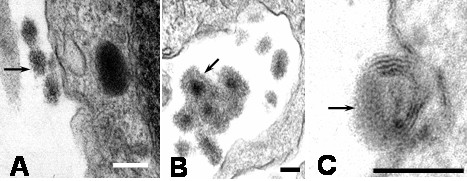
Electron microscope morphological observations of dense particles. A) Dense particles close to the cell surface (arrow; bar: 200 nm). B) Aggregated dense particles in the extracellular space (arrow). Note the nucleocapsid like center and the electron dense envelopes (bar: 100 nm). C) Dense particles showing a nucleocapsid like center surrounded by membrane layers and an electron dense material (arrow; bar: 100 nm).

**Figure 3:**
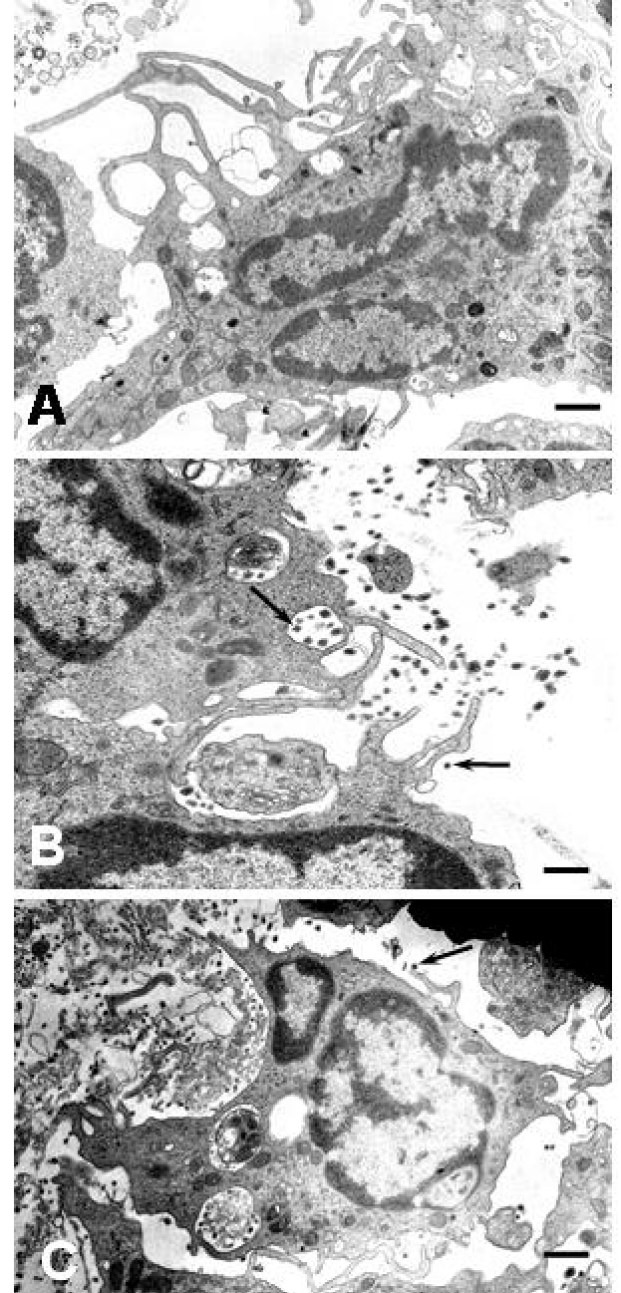
Ultrastructural features of DEN2 virus-infected monocytes. Prominent formation of cellular lamellipods (A) and engulfing of virus by macropicnocytosis (B) and phagocytosis (C) are observed 1 hour after infection. Note the presence of virus (arrows) and cellular debris in the extracellular space. (A and C bars: 1 μm; B bar: 500 nm).

**Figure 4:**
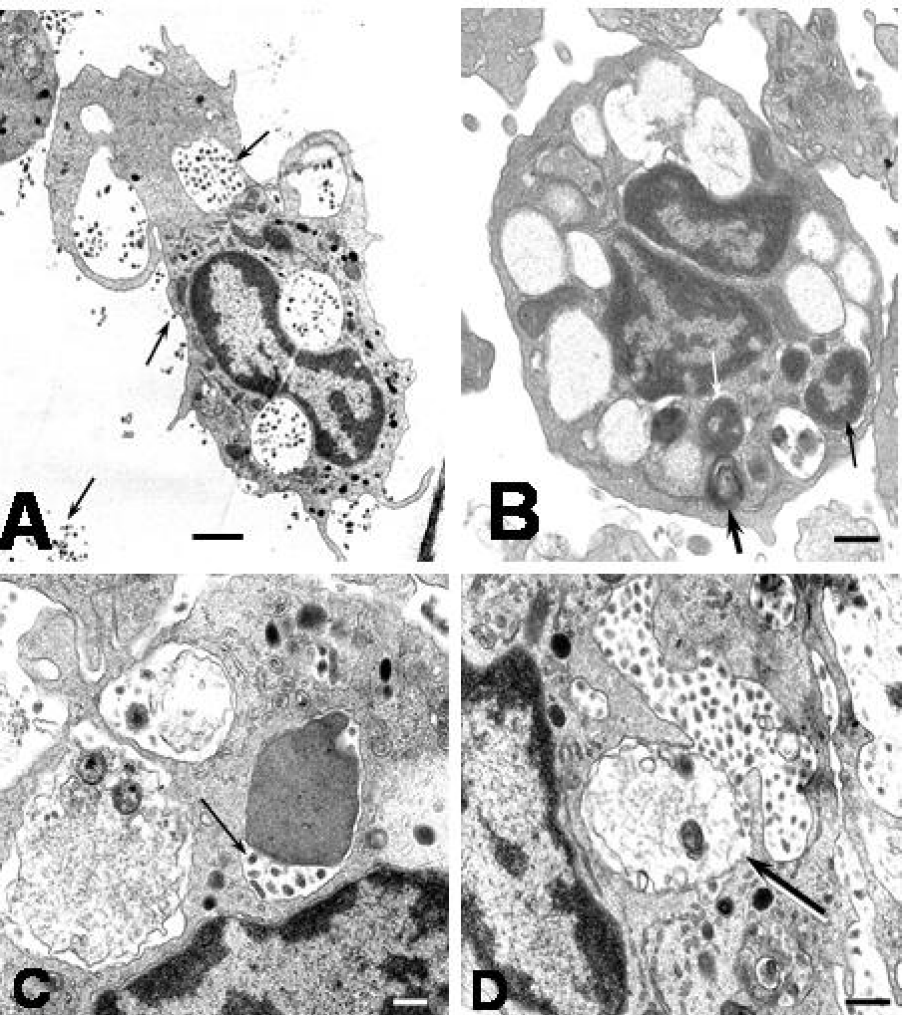
Ultrastructural features of DEN2 virus-infected monocytes. A) DEN2 virus-infected monocytes after 2 hours of infection. Observe the presence of virus particles in the extracellular space, on cellular plasma membrane and inside cytoplasmic vacuoles (arrows; bar: 1 μm). B) Monocyte showing huge empty vacuoles and vacuoles containing nuclear debris and myelin structures at 4 hours of culture (arrows; bar: 500 nm). C) Monocyte showing cytoplasmic phagosomes containing cellular debris and viral particles (arrow; bar: 200 nm). D) A huge vacuole containing numerous viral particles and cellular debris (arrow; bar: 500 nm).

After 1 hour of culture numerous virus particles were observed attached to plasma membrane, free in the extracellular space and in cytoplasmic vacuoles inside monocytes. The predominant viral particles in infected monocyte cultures were typical viral particles of 35 to 42 nm in diameter (Figures 1A, 1B, 1C). Small number of fuzzy coated viral particles (74 to 85 nm) showed a core similar to the usual dengue particles, but they had an envelope with projections, looking like a fuzzy coat (Figures 1D, 1E). Typical DEN2 virus particles observed in this study were similar to those reported in mosquito cell cultures [4]. Similar fuzzy coated virus particles have been described by Barth et al [4,5] in DEN2 Brazilian virus-infected C6/36 cell cultures. DEN2 virus used to infect monocytes was New Guinea C virus strain and isolated from virus-infected C6/36 cell cultures, suggesting that the fuzzy coated viral particles are a common feature of DEN2 virus. In addition, fuzzy coated virus particles have also been detected in other virus infections, but their significance remains obscure [6,7]. The presence of DEN2 virus antigens in the cytoplasm of infected monocytes was also investigated by direct immunofluorescence. Using a monoclonal antibody against DEN2 virus a diffuse and patchy patterns of fluorescence were observed in the cytoplasm (Figure 1F). It was also observed small electron dense structures (75 to 105 nm) that we called in this report "dense particles" (Figure 2). In some instances, these dense particles showed a center similar to dengue virus nucleocapsid covered by membrane layers and an electron dense envelope (Figures 2B, 2C). Dense particles could represent viral particles covered by a homogenous electron dense material. Since, it was not observe viral replication ultrastructural features in infected monocyte cultures, the contribution of monocytes to the formation of this viral envelope is unclear. However, electron dense material observed on the dense particles could represent a protein matrix obtained after virus replication on mosquito cells. In this regard, a range of variation in one virus after experimental isolation has been reported in other virus [6,7]. In general extracellular viral particles were found as single particles and viral particles forming aggregates were uncommon. Viruses attached to the cell surface and free in the extracellular space were engulfed by mechanisms of phagocytosis or macropicnocytosis via typical cytoplasmic processes (Figure 3). During phagocytosis or macropicnocytosis virus particles were engulfed alone or together with cellular debris, so that, intracytoplasmic vacuoles and vesicles containing viral particles or large phagosomes full of an electron dense matrix, cellular debris and viral particles may soon be found inside the cells (Figure 4). These data suggest a passive phase leading to virus inactivation. In this regard, previous reports have shown that human immunodeficiency virus entering human macrophages by phagocytosis is noninfectious [8]. Infection of Kupffer cells by dengue virus resulted in no viral progeny [9] and only a small proportion of the monocyte population supports replication of DEN2-virus [10]. Smooth membrane coated vacuoles containing viral particles, membrane fragments and moderated electron dense material were also observed (Figure 1B). In some instances, cytoplasmic vesicles containing one or more viral particles showed disruption of the membrane leading to direct communication of viral particles with the cytoplasm (Figure 1C), however, no morphological virus-related structures could be detected free in the cytoplasm. Features related to viral replication such as virus absorption by penetrating the cell membrane or by endocytosis by clathrin-coat vesicles, virion precursors on rough endoplasmic reticulum or its cisternae, inside Golgi complex, cytoplasm free viral cores or viral budding from cell membrane were not observed in monocytes. Previous report has shown that DEN2 virus can persistently infect transformed lymphoblastoid cells keeping an intact morphology without any indication of active viral replication [11]. Our data show no indications of viral replication and the induction of apoptosis (see below) makes monocytes unlikely source of persistent dengue virus infection.

### Monocyte cultures

**Figure 5:**
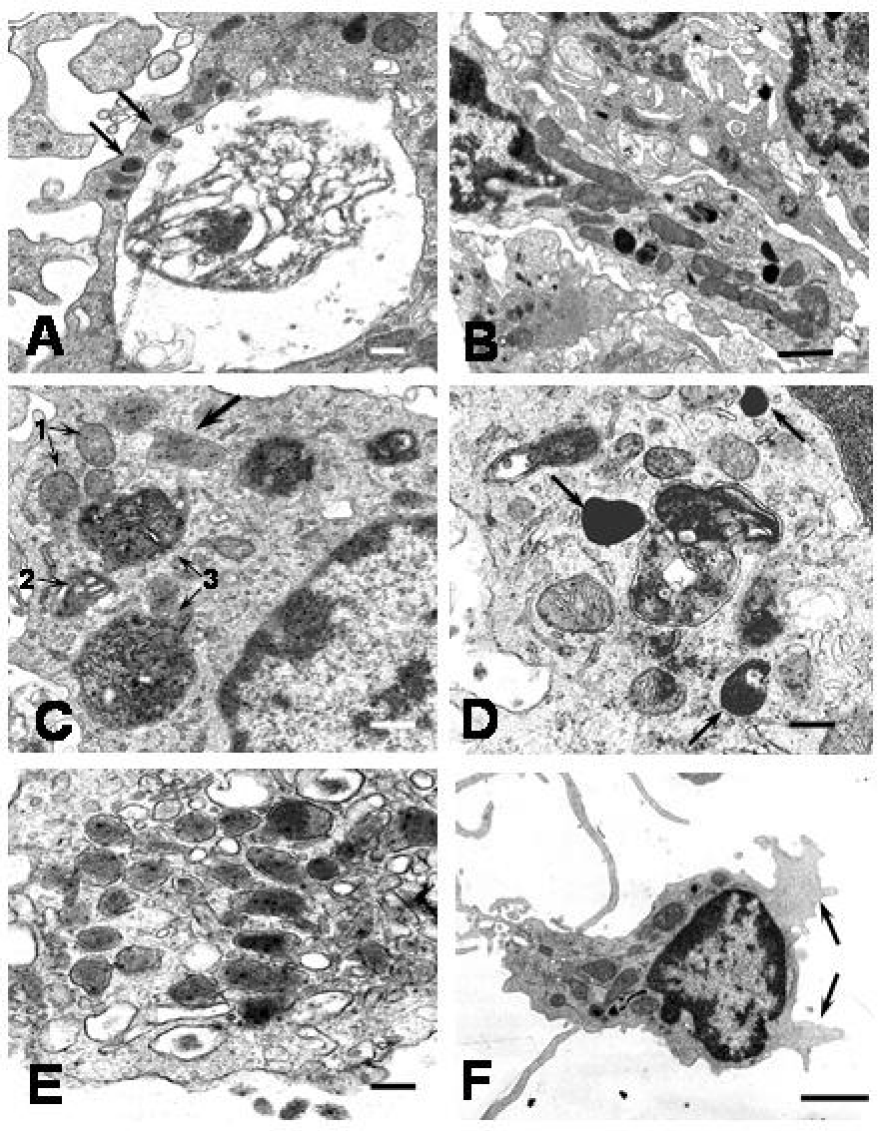
Ultrastructural features of DEN2 virus-infected monocytes at 4 hours. A) Cytoplasmic vacuole containing cellular debris in close association with lysosomal granules (arrows; bar 200 nm). B) Increased number and size of mitochondria in the cytoplasm of monocyte (bar: 1 μm). C) Mitochondrial degeneration: normal mitochondria (1), early step of degeneration (2) and late step of degeneration (3). Lysosomal granule (large arrow; bar: 500 nm). D) Lysosomes (arrows) in association with mitochondria an autophagosome containing probably mitochondrial debris (bar: 200 nm). Intense lysosomal and vesicular accumulation in the cytoplasm (bar: 200 nm). F) Leukocyte locomotion; note the formation of uropods (arrows; bar: 2 μm).

**Figure 6:**
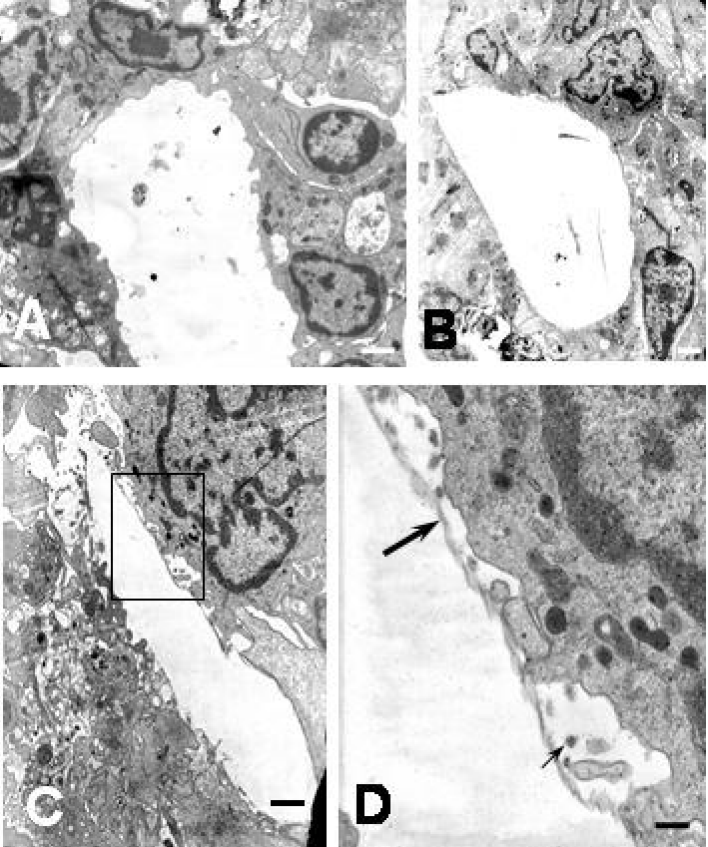
Ultrastructural features of DEN2 virus-infected monocytes. "Acinar" like structure. A and B show empty spaces surrounded by monocytes. (A bar 1 μm; B bar: 2 μm). C) In some instances, a moderated electron dense material also delimited the empty space (bar: 1 μm) D) Inset from C shows a lineal electron dense material (large arrow) delimiting the empty space, beyond viral particles (small arrow) and a monocyte are observed (bar: 200 nm).

As assessed by electron microscopy, monocytes showed high degree of activation after 1 hour of infection. One of the most prominent features in DEN2 virus-infected monocytes was the intense expression of short and long plasma membrane processes (lamellipods), in most of the cases engulfing virus particles, cellular debris and apoptotic cells (Figure 3). Engulfing of extracellular elements by pseudopods was also observed (Figure 3C). As consequence of this activity, small and huge intracytoplasmic vacuoles and phagosomes containing cellular debris, virus particles and myelin like structures in various stages of digestion were observed (Figures 3 and 4). In some instances, phagosomes or vacuoles were surrounded by lysosomes. (Figure 5A). Our data show similar ultrastructural findings than those obtained from DEN1 virus-infected Kupffer cells at 1 hour of culture [9], suggesting a similar cellular response against DEN virus for monocytes and macrophages. In DEN2 virus- infected monocytes mitochondria increased in number and size (Figure 5B) and cytoplasmic structures resembling diverse degrees of mitochondrial alterations (Figures 5C, 5D) were found. Mitochondria were observed in association with lysosomal granules and vacuoles containing membranous debris, consistent with mitochondrial digestion by lysosomes. Infected monocytes showed extensive proliferation of endoplasmic reticulum and lysosomal granules (Figure 5E). Cytoplasmic projections associated with cellular movement (uropods) were also observed (Figure 5F). It was not observed syncytia, however as shown in figure 6 a curious distribution of monocytes in DEN2 virus-infected cultures was found. Empty spaces were surrounded by monocytes looking like "acinar" structures. In some instances, a linear electron dense material occurred between the empty space and monocytes, suggesting a previous presence of biological material in the lumen. These findings could represent a reactive response of monocytes around virus particles, cellular debris or virus-infected cells.

### Cellular Death

**Figure 7:**
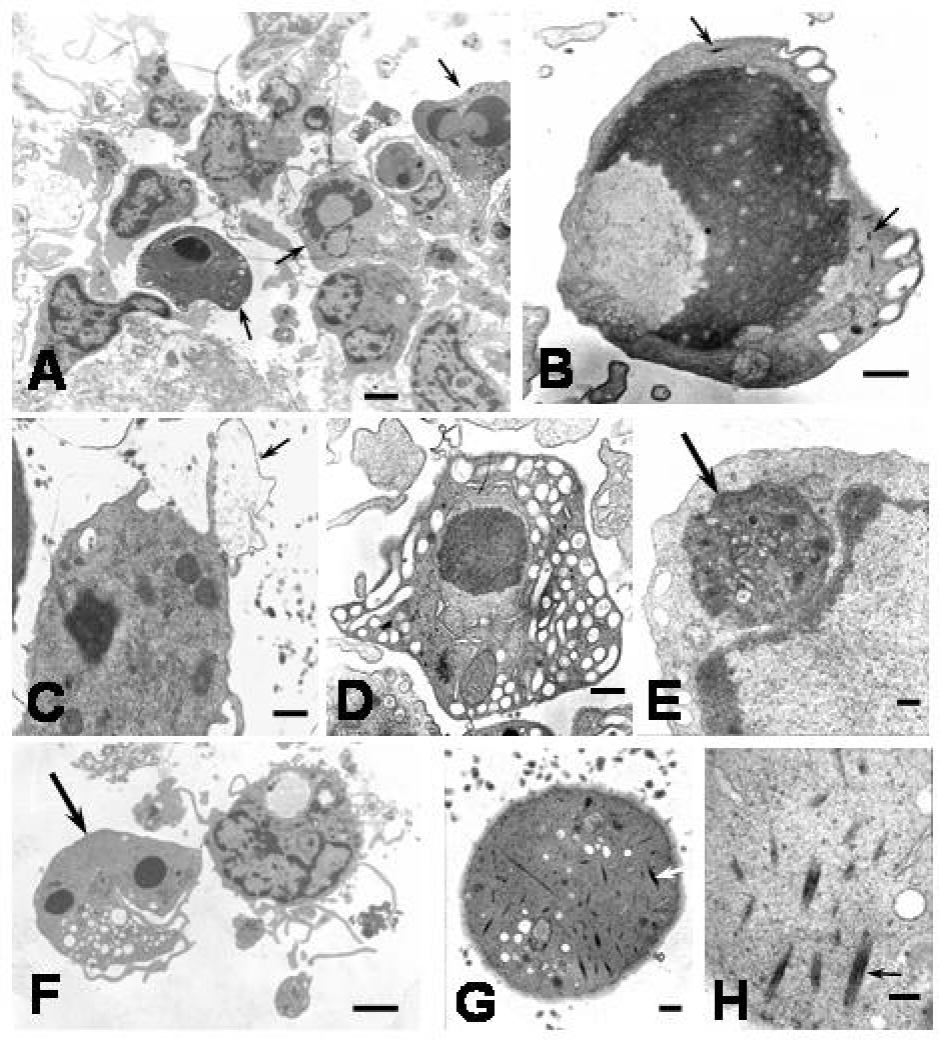
Ultrastructural features of apoptotic cells in DEN2 virus-infected monocytes at 4 hours. A) The typical features of apoptosis are observed in several monocytes (arrows; bar: 2 μm). B) Apoptotic cell showing cellular shrinkage, nuclear condensation and bundles of microfibrils (arrows; bar: 500 nm). C) Monocyte with dense remnant nucleus and surface blebbing (arrow; bar 500 nm). D) Apoptotic cell showing intense cytoplasmic vacuolization (bar: 500 nm). E) Phagosome in the cytoplasm of apoptotic cell (arrow; bar: 200 nm). F) Nuclear fragmentation in apoptotic cell (arrow). Note beside a healthy monocyte (bar: 2 μm). G) Segment of apoptotic cell showing numerous bundles of cytoplasmic fibrils (bar: 200 nm). H) Bundles of microfibrils (arrow) in the cytoplasm of apoptotic cell (bar: 100 nm).

**Figure 8:**
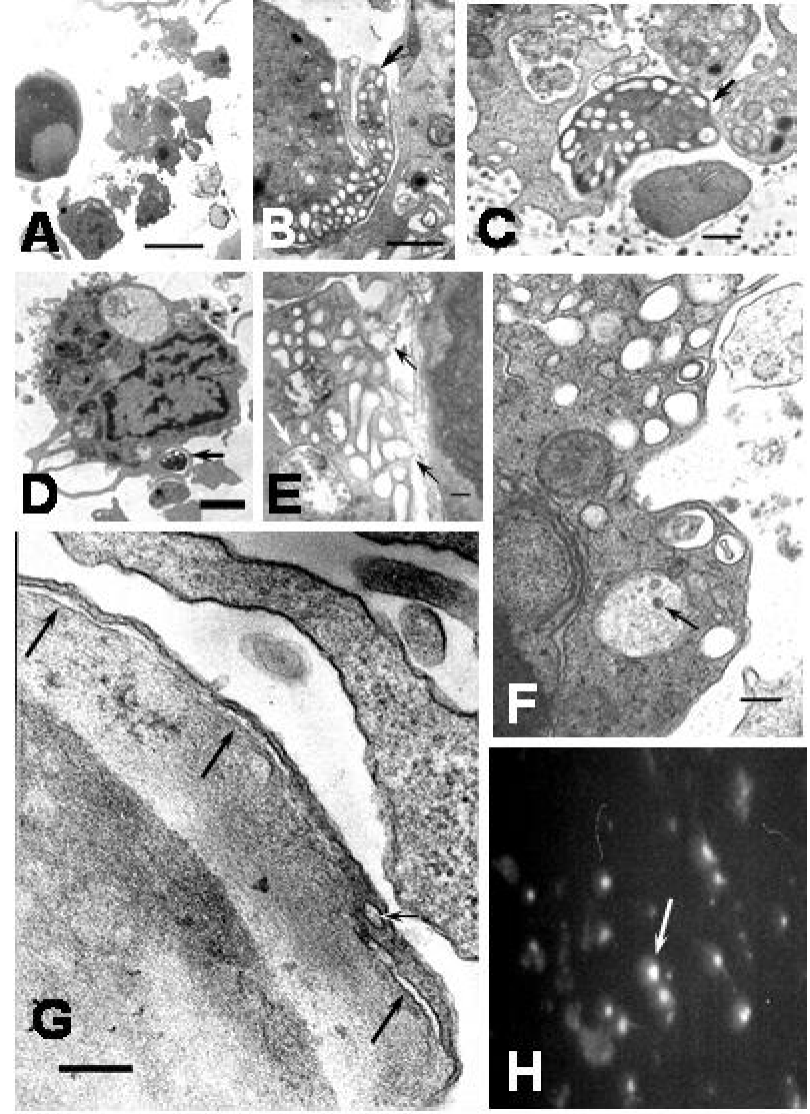
Ultrastructural features of apoptotic cells in DEN2 virus-infected monocytes. A) Apoptotic bodies containing nuclear fragments and several organelles (bar: 2 μm). B) Vesicular apoptotic body formation (bar: 1 μm). C) Vesicular apoptotic body in the extracellular space (arrow). Note a partial engulfing of the apoptotic body by monocyte processes (bar: 500 nm). D) Monocyte showing an engulfed vesicular apoptotic body (arrow) and intense accumulation of phagosomes containing cellular and viral material in several degrees of digestion (bar: 2 μm). E) Cytoplasm of apoptotic cell showing intense accumulation of vesicles and releasing of vesicular contents to the extracellular space (black arrows). Note the presence of a vacuole containing viral particles and electron dense material (white arrow; bar: 200 nm). F) Vacuole containing partial digested viral particles (arrow) in the cytoplasm of apoptotic cell (bar: 200 nm). G) Cisternae formation alongside the plasma membrane (arrows). Note a vesicle close to these formations (small arrow; bar: 200 nm). H) TUNEL staining for apoptosis in monocyte cultures infected for 4 hours with DEN-2 virus. Intense green fluorescence was observed in apoptotic nuclei (arrow). × 400.

**Figure 9:**
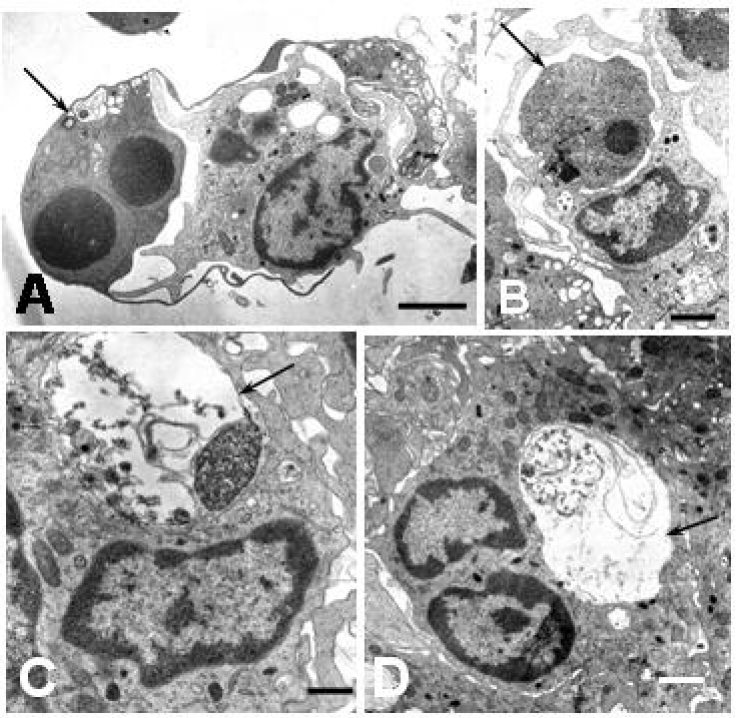
Ultrastructural features of apoptotic cells in DEN2 virus-infected monocytes. Different phases of phagocytosis and digestion of apoptotic cells. A) Engulfment of apoptotic cell (arrow) by a monocyte (bar: 2 μm). B) A huge phagosome containing a morphological intact apoptotic cell (arrow; bar: 1 μm). C and D show phagosomes (arrows) containing a partial digested apoptotic cells (C bar: 500 nm; D bar: 1 μm).

**Figure 10:**
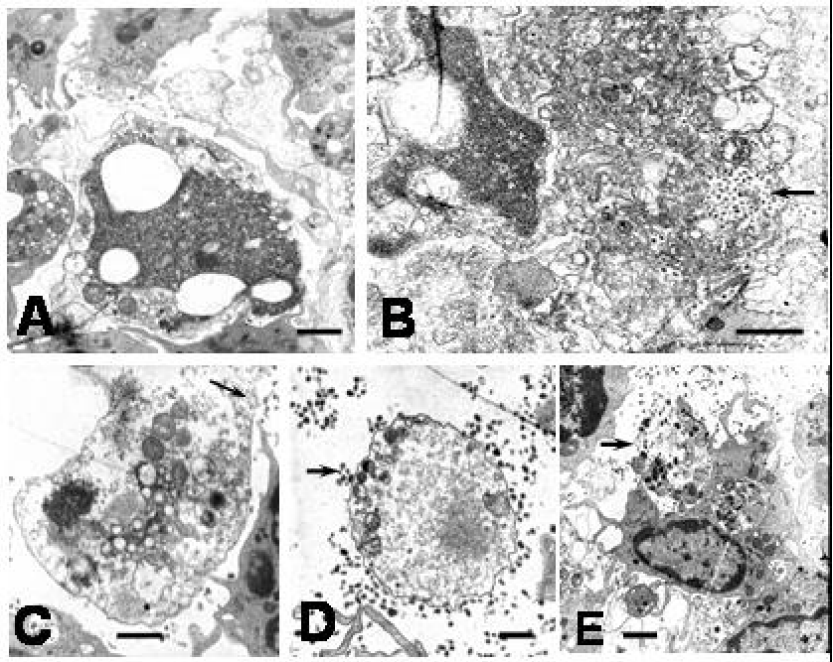
Ultrastructural features of apoptotic cells in DEN2 virus-infected monocytes at 6 hours. A) Swelling of organelles and membrane compartments in an apoptotic cell (bar: 1 μm). B) Release of cellular content from a swelling apoptotic cell. Note numerous viral particles (arrow) probably already present in the extracellular space (bar: 1 μm). C) Advance phase of cellular swelling (ghost cell) showing disruption of plasma membrane (arrow; bar: 1 μm). D) Ghost cell surrounding by numerous viral particles (arrow; bar: 500 nm). E) Monocyte engulfing cellular debris (arrow). Note the presence of a phagosome containing partial digested cellular material (bar: 1 μm).

After 1 hour of infection, electron microscopy revealed cells with morphological features of apoptosis, however, previous report has shown apoptosis in Kupffer cells [9] after 24 hours of DEN-1 virus infection, suggesting different susceptibility of monocyte and macrophage to virus-induced apoptosis or different viral apoptotic effect depending of DEN virus strain. In this regard, the susceptibility to DEN virus infection depending of the differentiation state of monocytic cells has been reported [12]. Apoptotic cells showed chromatin margination in nuclei, nuclear fragmentation, condensation and retraction of cytoplasm and blebbing and budding phenomena (Figures 7 and 8). Numerous vesicles, some of which appeared to be releasing to the extracellular space were observed (Figures 7D and 8E). The budding phenomenon observed on apoptotic cells led to the formation of apoptotic bodies containing several types of organelles, including nuclear fragments and high number of vesicles. This could represent a common aspect in virus-induced apoptosis, since the formation of vesicular apoptotic bodies has also been reported in monocytic/macrophage lineage infected with bovine leukaemia virus [13]. Blebbing of the plasma membrane was also observed in apoptotic cells. The surface blebbing has also been described in other viral infections and related to a role in the direct cell-to-cell spread of the virus [14] or associated with increased cellular permeability [15]. Some apoptotic cells showed long cisternae structures alongside with the plasma membrane suggesting cytoplasmic splitting (Figure 8G). We have no explanation for this finding, but it could be due to the fusion of neighboring cytoplasmic vesicles. Apoptotic cells also showed bundles of intracellular microfibrils (Figures 7G and 7H), which resembled the contractile structures observed in fibroblasts and some glomerular cells [16]. These structures could be related to the apoptotic process, since, filamentous material, clumping of tonofilaments and MyD88 protein association with fibrillar aggregates containing beta-actin have been associated with apoptosis and apoptotic bodies formation [17-19]. Huge phagosomes were observed in the cytoplasm of apoptotic cells (Figure 7E), and in some instances, vacuoles containing few viral particles associated with an electron dense material were observed (Figures 8E and 8F). The presence of phagosomes in the cytoplasm of apoptotic cells suggests previous active phagocytosis. Contrarily to non apoptotic cell only scarce number of vacuoles containing virus and degraded material was observed in apoptotic cells, suggesting that the absorption of products of viral degradation could trigger cell death. Several apoptotic monocytes and apoptotic bodies were ingested by neighboring healthy monocytes leading to the formation of huge vacuolar compartments containing different grades of cellular digestion (Figures 8D and 9). Apoptosis could avoid the release of viral particles [20] and together with the phagocytosis and digestion of apoptotic cells represent mechanisms to prevent viral progeny [9,21,22]. The ultrastructural apoptosis finding was confirmed by detecting intrachromosomal DNA strand breaks using the TUNNEL assay. Untreated cultures showed low levels of TUNEL positive cells compared to higher levels observed in infected monocyte cultures (Control: 0.9 ± 0.15. Infected at 1 h: 6.2 ± 1.5; 2 h: 6.4 ± 1.8; 4 h: 7.4 ± 2.3; 6 h: 16.8 ± 3.3; mean ± SE) (Figure 8H). In addition to apoptosis, a cellular alteration accompanied by cellular swelling, plasma membrane disruption and karyolysis was observed (Figure 10). Plasma membrane disruption led to increased amount of swelled organelles and cellular debris in the extracellular space and the formation of "ghost cells" (Figures 10C and 10D), with further engulfing by monocytes (Figure 10E). These lysed cells could represent nonphagocytized apoptotic cells that have lost the membrane integrity [23]. Since, noninfected controls or heat-inactivated DEN2 virus-infected monocytes showed scarce number of apoptotic cells, apoptosis seems to be linked to virus infection. We can not rule out the role of apoptosis inducer proteins in the apoptosis observed in this study. In this regard, increased production of Tumor Necrosis Factor has been reported in DEN2 virus-infected macrophages which could lead to apoptosis. [24].

## Conclusion

This *in vitro *study indicates that the interaction of DEN2 virus with monocytes results in virus engulfment and apoptosis, suggesting that monocytes may protect against DEN2 virus infection by eliminating the virus particles and virus-infected apoptotic cells and this could be important in the rapid clearance of the initial virus input.

## Methods

### Preparation of virus stock and virus titration

DEN-2 virus strain New Guinea C was propagated in C6/36HT mosquito cells that were cultured in Eagle's MEM medium containing 10% FBS prior to viral monocyte infection. The virus culture medium was harvested after 5 days of incubation and after removal of cell debris by centrifugation, the virus supernatant was aliquoted and stored at -70°C until used. Virus was titrated by plaque formation assays on VERO cells. Cells were planted at 1 × 10^6 ^cells / well in 24-well plates and subsequently, serial dilutions of virus were added and the mixtures were incubated at 37°C for 7 days. Afterwards, the plaques were visualized by staining with a dye solution composed of 1% crystal violet. Virus concentrations are given as plaque-forming units (PFU) / ml. Virus stock was free of endotoxin as determined by limulus amebocyte lysate assay.

### Monocyte cultures

Monocytes were isolated from heparinized peripheral blood obtained from human healthy volunteers (N = 5) by density centrifugation over 1.077 Histopaque (Sigma Chemical Co, St. Louis, MO). Healthy individuals were informed about the study procedures and their consents were obtained before enrollment in the investigation following the ethical committee guidelines of the bioethical committee of Medical School (Universidad del Zulia, Maracaibo, Venezuela). Total mononuclear leukocytes recovered from the interface were washed and resuspended in RPMI 1640, 10 % fetal bovine serum and penicillin/streptomycin. Afterwards, 300 μl / well of a cellular suspension (4 × 10^6 ^cells / ml) were layered on 8 -well plastic chamber slides (Nunc, Roskilde, Denmark) or 10 ml on 75 cm^3 ^tissue culture flasks and incubated for 3 hours at 37°C and 5% CO_2_. Non adherents cells were washed out with warm medium and adhered cells were used for experiments.

### Infection of monocyte cultures

Monocytes were infected with a virus concentration of 4 × 10^4 ^PFU / ml (MOI: 0.08) and incubated for 1, 2, 4 and 6 hours at 37°C and 5% CO_2_. Controls represent monocytes cultured with supplemented medium without virus. In addition, monocyte cultures were incubated with heat inactivated dengue virus (56°C, 30 min.) at 4 × 10^4 ^PFU / ml for 6 hours.

### Electron microscopy studies

Monocytes planted on 75 cm^3 ^tissue culture flasks were incubated for 1, 2, 4 and 6 hours with DEN-2 virus (4 × 10^4 ^PFU/ml). Afterwards, cells were detached by incubation with a solution of 0.01% EDTA and by using a cell scraper. After centrifugation, infected monocytes and controls were fixed with 2% glutaraldehyde in 0.1 M cacodylate buffer, pH 7.3. Cells were postfixed with 1% osmium tetraoxide, dehydrated in a series of ethanol and embedded in Epon 812. Samples were cut into ultrathin sections, stained with uranyl acetate followed by lead citrate and examined in an electron microscopy JEM 1010 (Jeol, Japan).

### Direct immunofluorescence for DEN-2 antigens

Experiments were performed in 8-well plastic chamber slides. Monocytes were infected by incubation with DEN-2 virus as described above. Monocytes were washed in PBS and fixed with cold acetone for 5 minutes. Intracellular viral antigens were detected by a direct immunofluorescence assay using a fluorescein-conjugated DEN-2 virus-specific monoclonal antibody (CDC, Fort Collins, CO. USA).

### TUNNEL assay

The method for nick end -labeling of apoptotic cells was adapted from that of Gavrieli et al. [25] with a commercial kit (Pharmigen, San Diego, CA). Adhered monocytes were treated according to the protocol provided with this kit. The assay is based on the preferential binding of the FITC-dUTP by terminal deoxynucleotidyl transferase to 3' OH ends of the DNA. Positive apoptotic nuclei were assessed by fluorescence microscopy (Axioskop, Zeiss, Germany).

## Competing interests

The authors certify that they have not entered into any agreement that could interfere with their access to the data on the research, or upon their ability to analyze the data independently, to prepare manuscripts, and to publish them. Authors have not any conflicts of interest.

## Authors' contributions

JM designed, coordinated and draft the manuscript. JPH performed the ultrastructural procedures. NV, LME, GA performed TUNEL assay, virus isolation, monocytes cultures. All authors read and approved the final manuscript.
